# The Impact of Ecological Niche on Adaptive Flexibility of Sensory Circuitry

**DOI:** 10.3389/fnins.2017.00344

**Published:** 2017-06-28

**Authors:** Sarah L. Pallas

**Affiliations:** Neuroscience Institute, Georgia State UniversityAtlanta, GA, United States

**Keywords:** sensory deprivation, cross-modal plasticity, topographic maps, synaptic plasticity, inhibitory plasticity

## Abstract

Evolution and development are interdependent, particularly with regard to the construction of the nervous system and its position as the machine that produces behavior. On the one hand, the processes directing development and plasticity of the brain provide avenues through which natural selection can sculpt neural cell fate and connectivity, and on the other hand, they are themselves subject to selection pressure. For example, mutations that produce heritable perturbations in neuronal birth and death rates, transcription factor expression, or availability of axon guidance factors within sensory pathways can markedly affect the development of form and thus the function of stimulus decoding circuitry. This evolvability of flexible circuits makes them more adaptable to environmental variation. Although there is general agreement on this point, whether the sensitivity of circuits to environmental influence and the mechanisms underlying development and plasticity of sensory pathways are similar across species from different ecological niches has received almost no attention. Neural circuits are generally more sensitive to environmental influences during an early critical period, but not all niches afford the same access to stimuli in early life. Furthermore, depending on predictability of the habitat and ecological niche, sensory coding circuits might be more susceptible to sensory experience in some species than in others. Despite decades of work on understanding the mechanisms underlying critical period plasticity, the importance of ecological niche in visual pathway development has received little attention. Here, I will explore the relationship between critical period plasticity and ecological niche in mammalian sensory pathways.

“…evolution is the control of development by ecology.”

-*Leigh van Valen*

## Development both facilitates and constrains adaptation

Early events in nervous system development are very similar across species because they provide a basic framework upon which more species-specific events are built at later time points. Mutations that affect early events are likely to be deleterious or even lethal, and thus they place severe constraints on potentially adaptive variation. If they are not deleterious, early changes could produce profound alterations in structure and function, affecting any circuitry that is dependent on that early framework. Mutations that occur later in nervous system development would have less of an effect, but because of the interconnected nature of neurons, even small changes in one member of a network will affect all members of the network. This is a potentially dangerous situation, and thus evolution has come up with work-arounds that can preserve neural network function despite the unavoidable missteps that can occur in brain building. Many of those work-arounds involve built-in flexibility that allows networks to adapt to variation within a lifetime as well as across evolutionary time, thus facilitating adaptation.

## Target specificity

One of the most critical steps in building neural circuits is for axons to locate and make synapses within the proper target. At one extreme, each axon could have its target choices pre-specified. This was the premise behind Sperry's chemoaffinity hypothesis. When he cut the optic nerve and rotated the eye of a frog, the axons within the optic nerve regenerated and made synapses with their original target sites in the optic tectum, leading to frogs that made 180° errors in locating visual stimuli. These results suggested to Sperry that there is a chemical address system in which axons and targets have matching labels that they use to find each other in a proverbial haystack.

Sperry's findings suggested that evolution had provided a strict one to one wiring diagram for the brain. What Sperry didn't realize is that frogs can eventually make corrections in their retinotectal wiring, correcting their visual localization ability. Similarly, Xenopus tadpoles, which have binocular vision as a result of the intrahemispheric connections of the nucleus isthmi, can realign those connections after eye rotation (Udin and Keating, 1981; Udin, 2012, for review). In an extreme example, a third eye primordium transplanted onto a tadpole's head can successfully compete with the existing eyes for target space in the optic tectum. The extra eye drives the formation of eye-specific termination regions that resemble the ocular dominance stripes seen normally in binocular visual cortex of carnivores and primates (LeVay et al., 1978, 1980; Law and Constantine-Paton, 1981). In contrast to Sperry's more rigid chemoaffinity hypothesis, these findings illustrate the power of visual experience to guide not only normal connectivity patterns between eye and brain, but to compensate for unique circumstances in a way that optimizes function.

When initially considered the corrections to retinotectal maps in frogs seem quite remarkable. However, the wiring of input and target neurons is normally shaped by experience to some extent. The “fire together, wire together” and “use it or lose it” principles of Hebbian learning (Hebb, 1949) can account for experience-dependent changes in the strength and maintenance of synaptic connections. NMDA receptors allow activity levels to be translated into synaptic strength (Constantine-Paton and Cline, 1998). Even before eye opening, spontaneous activity that resembles visually driven activity occurs at several points within the visual pathway (Meister et al., 1991; Weliky and Katz, 1999; Chiu and Weliky, 2001) and can guide normal circuit wiring to a considerable extent. This is important when considering that the point at which birth and eye opening occur with respect to gestation varies across species. Thus, in more altricial, nocturnal, and fossorial species, spontaneous activity may be a more important factor than in precocial, diurnal, cursorial species in shaping connectivity relative to vision.

## Cross-modal plasticity

**Figure 1:**
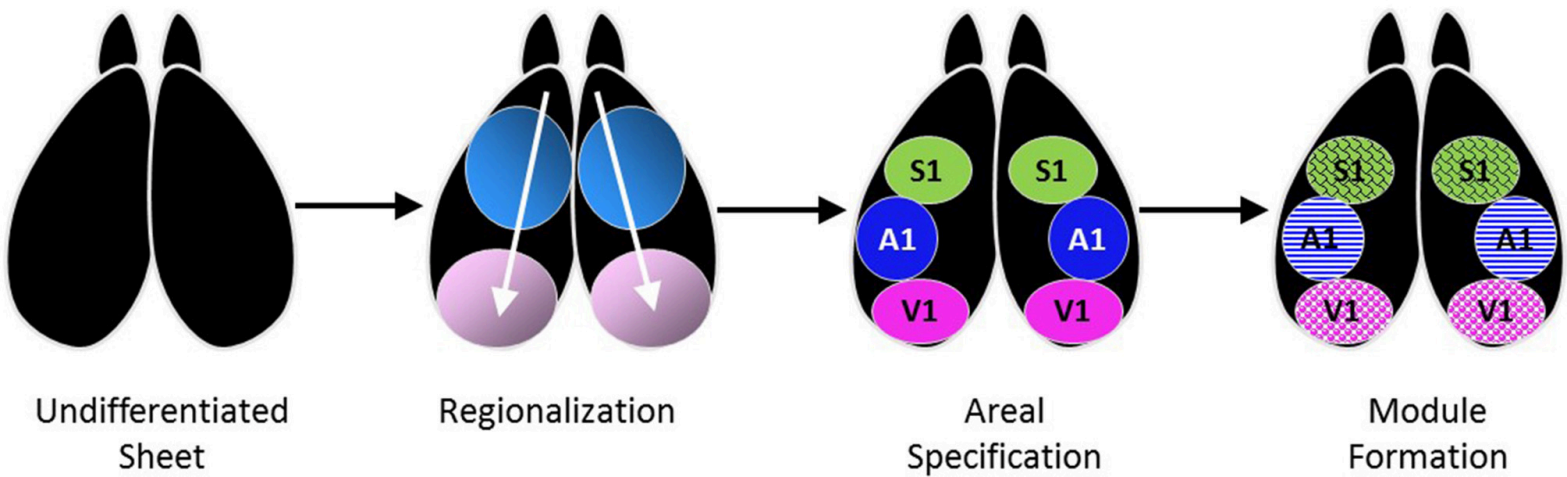
Specification of primary sensory areas in cerebral cortex occurs gradually, starting with establishment of positional identity and polarity gradients that lead to regionalization, followed by formation of strict boundaries between cortical areas and the thalamocortical projections that bring in sensory information. Within each area, modules for special processing are distributed in a pattern that is characteristic for each area (Modified from Pallas et al., 2006, used with permission).

Another illustration of the extent to which axons can be flexible in their target choice comes from studies of cross-modal plasticity in sensory cortex. Cerebral cortex develops in a stepwise fashion (Figure 1), beginning from an undifferentiated, laminated sheet with common features throughout. Regional information is established under the control of various transcription factors and morphogens, some of which are arranged in opposing gradients (Puelles and Rubenstein, 2003; Ypsilanti and Rubenstein, 2016). How precise boundaries form between adjacent cortical areas is not well-understood. The formation of area-specific modules, such as cytochrome oxidase blobs and ocular dominance columns in primary visual cortex (V1) (LeVay et al., 1978; Trusk et al., 1989), binaural bands in primary auditory cortex (A1) (Middlebrooks et al., 1980), and whisker barrels in primary somatosensory cortex (S1) (Woolsey and Van der Loos, 1970), occurs under the partial direction of neural activity. Studies of cross-modal plasticity investigate the extent to which these area-specific features are flexible.

**Figure 2:**
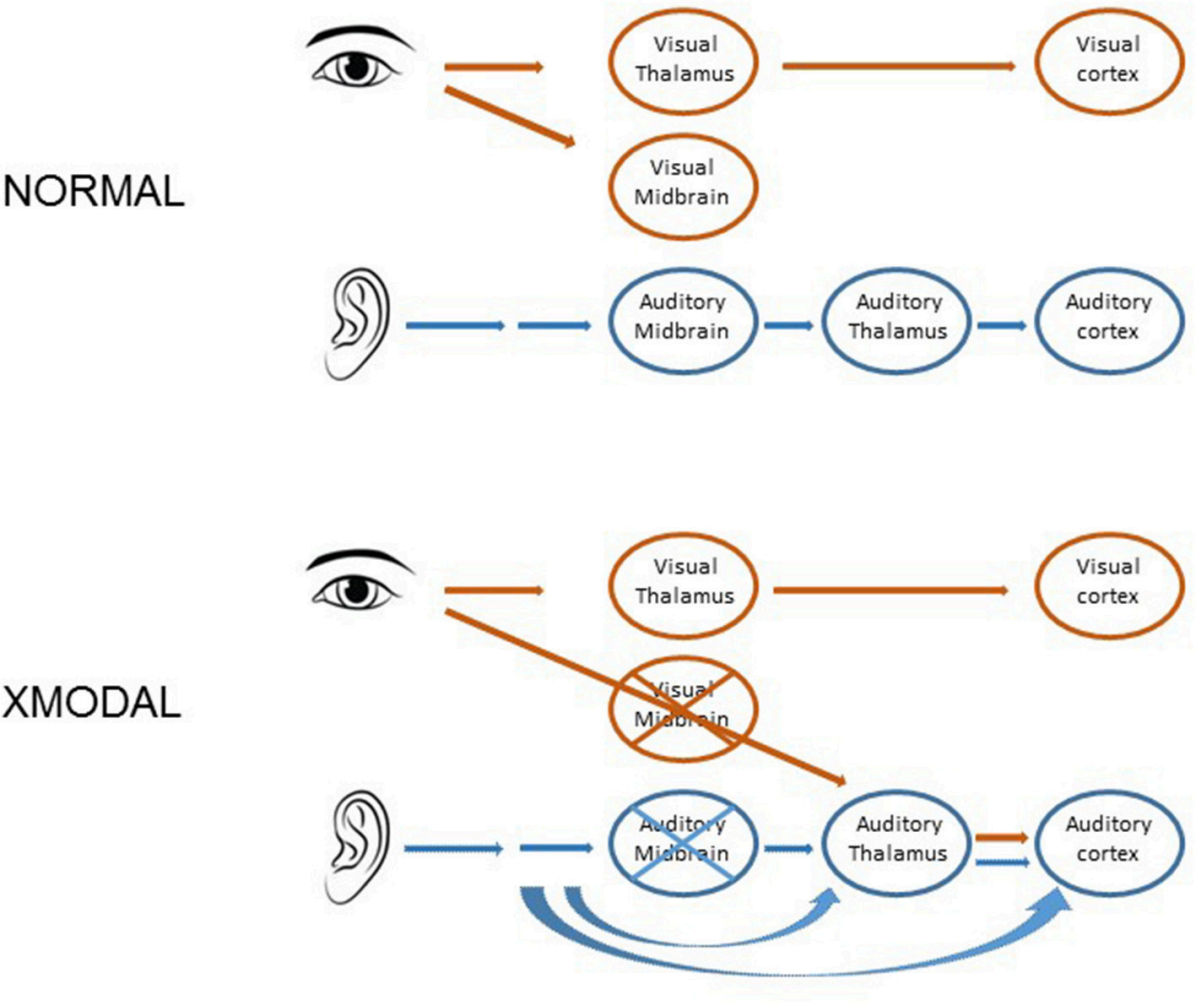
Cartoon depicting the normal projections in the auditory and visual pathways **(top)** and the surgical procedure that leads to cross-modal plasticity in ferrets **(bottom)**. As a result of the procedure, the retina invades the auditory thalamus, which in turn conveys visual activity to the auditory cortex.

**Figure 3:**
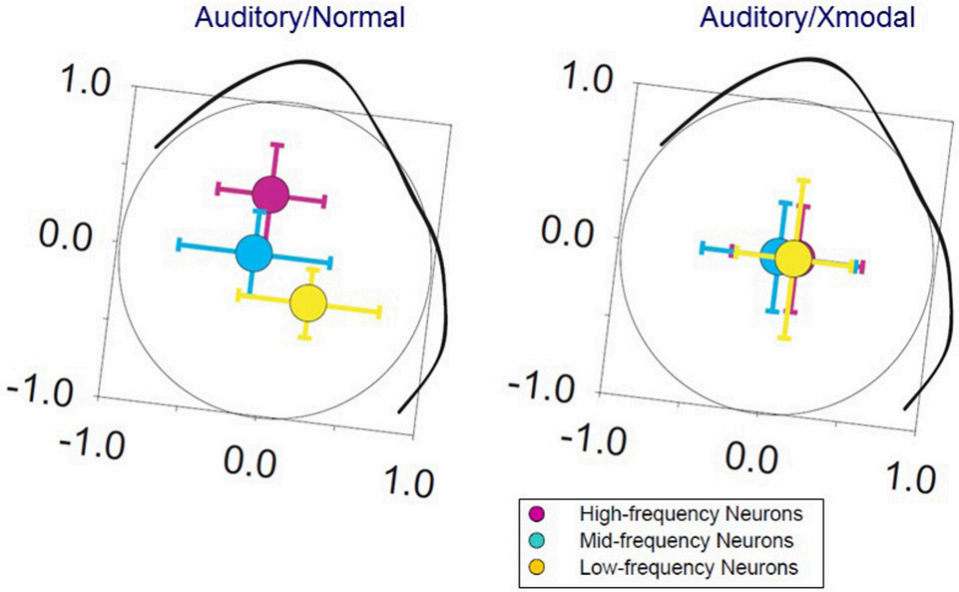
Tonotopic maps in normal ferrets are oriented such that high frequencies are represented medially and low frequencies laterally **(left)**. In auditory cortex of ferrets in which retinal axons have invaded auditory thalamus, visually-responsive, sound-responsive, and bisensory neurons are intermixed **(right)**. The tonotopic map in these ferrets is absent, with no significant difference in the spatial center of distribution (colored circles) of high, medium, or low-frequency tuned neurons (error bars indicate ± standard error. Modified from Mao and Pallas, 2012, used with permission).

In hamsters, mice, and ferrets, neonatal damage to the sensory midbrain, which reduces retinal target space and deafferents some sensory thalamic regions, can induce retinal axons to invade non-visual targets, including the auditory thalamus and the somatosensory thalamus (Schneider, 1973; Frost, 1982; Sur et al., 1988; Ellsworth et al., 2005). In ferrets, midbrain damage results in a partial takeover of auditory thalamus and auditory cortex by visually driven activity (Figure 2). The circuitry within auditory cortex is altered in response, such that auditory cortical responses to light stimuli resemble those in visual cortex, including the presence of a two-dimensional map of visual space (Sur et al., 1988; Roe et al., 1990, 1992; Pallas and Mao, 2012, for review). Callosal and local connectivity patterns were altered and reorganized in a way that suggested a splitting of the auditory cortical area into segregated auditory and visual subareas (Gao and Pallas, 1999; Pallas et al., 1999). To the contrary, we discovered that although auditory responses remain, tuning to sound frequency is broader, the tonotopic map is virtually absent (Figure 3), and sound-responsive neurons have higher thresholds in cross-modal auditory cortex, perhaps due to changes in organization of inhibitory interneurons (Mao et al., 2011b; Mao and Pallas, 2012, 2013). In addition, multisensory neurons that respond to either sound or light stimulation are created at the expense of sound-only neurons. The number of visual-only neurons increases with the extent of the early damage. These results show that, although the cerebral cortex is quite flexible in its ability to accommodate various types of inputs, there is a limit to the ability to do two things at once, at least in primary auditory cortex. The difficulty may be one of topography. In multisensory cortical regions that do successfully represent two modalities, they share a common topographic basis—such as location of an auditory or visual stimulus in space (Wallace et al., 1992, 2006). In primary auditory cortex, there is no map of stimulus location; rather it contains a map of sound frequency. The two dimensional map of visual space created in cross-modal primary auditory cortex (Roe et al., 1990) may interfere with the one-dimensional map of frequency, and thus with a major organizing principle of A1, leading to the degradation of tuning that we observed.

Cross-modal plasticity may seem an extreme response to loss of input or target space only obtained through special experimental circumstances. This is far from being true; cross-modal plasticity in the form of sensory substitution occurs both evolutionarily and clinically. Animals with evolutionarily reduced visual input, such as blind cave fish (Hinaux et al., 2016) or blind mole rats (Heil et al., 1991; Bronchti et al., 2002) exhibit a takeover of the underutilized visual regions by other senses. On a developmental time scale, in deaf or blind animals including humans, the intact sense takes over territory that would normally belong to the deprived or damaged sense. This produces what can seem like supernatural ability in the intact sense (Rauschecker et al., 1992; Rauschecker and Korte, 1993; Bavelier and Neville, 2002; Lomber et al., 2010; Butler et al., 2017; Glick and Sharma, 2017; Kral et al., 2017; Schormans et al., 2017). The ability of sensory cortex to reconfigure its organization and connectivity according to unforeseen circumstances would predispose it to adapt to evolutionary change (Pallas, 2007; Kral and Pallas, 2011; Pallas and Mao, 2012, for review).

## Population matching and cell death

Another way in which developmental mechanisms can predispose circuits to accommodate new afferents, allow innervation of new target space, and compensate for changes in either population is through flexible population matching mechanisms. Many more neurons are generated in early development than survive until adulthood, and survival of afferents can be affected by availability of target space (Hamburger and Levi-Montalcini, 1949; Hollyday and Hamburger, 1976). The reverse is also true; target neurons are dependent upon innervation for survival (Pallas et al., 1988; Buss et al., 2006). Furthermore, the interconnectedness of brain pathways means that a change in number of neurons in one region will affect all members of the pathway like a stack of dominoes. The evolutionary benefit is that a mutation that increases or decreases the number of neurons at one locus of a pathway will be accommodated through changes in neuron survival or alterations in branching at every level of the pathway.

The more connectivity options that a neuron has, the less it will be affected by a decrease in target size (Finlay and Pallas, 1989, for review). For example, retinal axons have many potential targets, and if one is lost, the axons will increase their projections to alternate targets, even to other modalities (Figure 2). On the other hand, some brain regions receive input from or send inputs to only a single other region. One example is the lateral geniculate nucleus (LGN), which requires primary visual cortex (V1) in order to survive. Ablation of V1 leads to massive cell death in LGN (Raabe et al., 1986; Woo et al., 1992), but ablation of large portions of thalamus has little impact on cerebral cortex (Miller et al., 1991) due to the many alternate synaptic partners for cortical neurons. From an evolutionary perspective, singly targeted afferent populations seem risky. One might speculate that the cost is lower than the benefit of having a dedicated communication channel between sensory thalamus and primary sensory cortices.

## Population matching in topographic maps

Whether a decrease in target size affects function has been addressed in studies of topographic map compression. In adult frogs and fish, ablation of the caudal half of the optic tectum results in a compression of the regenerating retinal axons onto the remaining half (Udin, 1977; Schmidt, 1983). Although, the optic nerve in adult mammals does not regenerate without heroic efforts (Bei et al., 2016; Lim et al., 2016), it can regenerate in neonatal hamsters (Finlay et al., 1979) and mice (Pallas, in preparation). Map compression in neonatal hamsters occurs without substantial increases in retinal cell death (Wikler et al., 1986), such that a 50% lesion of the superior colliculus (SC) leads to a doubling of the input/target ratio. Remarkably, this occurs without a concomitant increase in SC neuron receptive field size (Pallas and Finlay, 1989). The preservation of receptive field size is achieved by a reduction in retinal axon arbor complexity and by a selective redirection of some retinal axons to alternate target regions (Pallas and Finlay, 1991; Xiong et al., 1994). This result implies that the SC neurons have a way to recognize how much visual space is represented by the retinal ganglion cells competing for target space. Thus, despite having twice as many retinal afferents available to them, SC neurons select only those that represent the same amount of visual space as in normal, non-compressed maps. We tested the hypothesis that, although the compression itself is activity-independent, NMDA receptors on the SC neurons could provide a filter for the degree of receptive field overlap of the competing retinal inputs. Chronic blockade of NMDA receptors in SC during post-natal development prevented the normal refinement of receptive fields, as seen in other species (Debski et al., 1990; Schmidt et al., 2000). It also blocked the compensation process for map compression, leading to receptive fields within the compressed maps that were even larger than in normal juveniles (Huang and Pallas, 2001), supporting the hypothesis.

The changes in axon arbor complexity might be expected to degrade stimulus tuning. As in the “bug detector” neurons in frog optic tectum (Lettvin et al., 1959), neurons in superficial SC of rodents are tuned to stimulus size and velocity, preferring small, slowly moving objects (Razak and Pallas, 2005, 2006). In animals that have undergone map compression, stimulus size tuning, and stimulus velocity tuning of the population of SC neurons are normal (Pallas and Finlay, 1989). NMDA receptor blockade had no effect on size or velocity tuning (Huang and Pallas, 2001; Razak et al., 2003). Instead, an increase in the strength and spatial extent of lateral inhibition in compressed maps apparently compensates for the excess retinal inputs in a way that preserves stimulus tuning properties (Razak and Pallas, 2007; Razak et al., 2010). That receptive field properties remain stable even for massive changes in afferent/target ratios makes a powerful argument that developmental mechanisms can predispose the brain to accommodate evolutionary changes in neuron population numbers.

**Figure 4:**
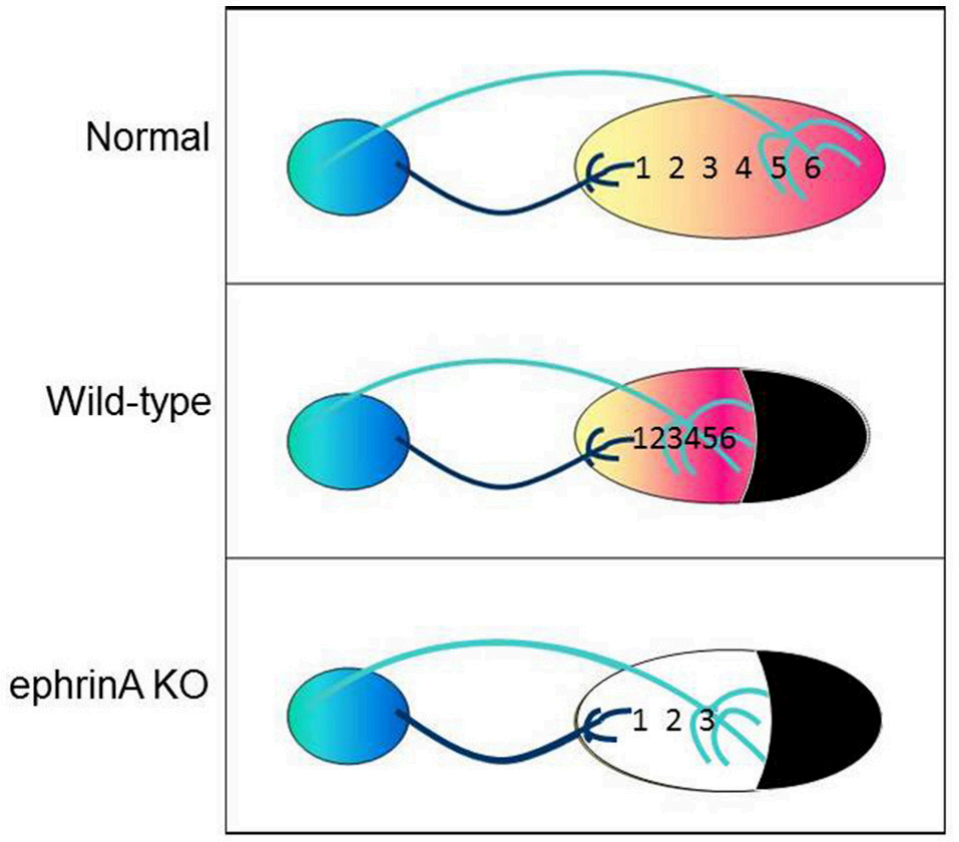
Cartoon illustrating the mapping of nasal to temporal visual field locations 1–6 onto the anterior to posterior axis of SC in normal animals **(top)**, in animals with compression of the entire visual field onto the smaller SC after neonatal ablation of caudal SC **(middle)**, and in ephrin knockout animals that failed to compress their retino-SC projections **(bottom)**.

Given that gradients of the repulsive guidance factors ephrin-A2 and -A5 in the SC and their EphA receptors in the retina are responsible for setting up the topographic map in normal SC (Feldheim et al., 2000, 2004; Cang et al., 2005), we reasoned that they might also be responsible for map compression. Our correlative gene expression study supported this hypothesis; SC size after neonatal lesion correlates not only with the steepness of the retino-SC map, but also with the steepness of the ephrin-A2 and eprhin-A5 gradients (Tadesse et al., 2013). Preliminary data with ephrinA knockout mice (kindly donated by David Feldheim and Renping Zhou) are consistent with the hypothesis that ephrinAs are necessary for the retino-SC maps to compress (Mao et al., 2011a, and in preparation; Figure 4). Whether the early damage to SC that triggers the map compression is first triggering the redistribution of ephrin-As or vice versa is unknown, and is currently under study. At any rate, regulation of guidance cue distribution by the size of the brain region would be another developmental process that could accommodate evolutionary change in an adaptive way.

## Receptive fields are the currency that sensory neurons use to represent the stimulus space

The “classical” receptive field (RF) derives from the spatiotemporal sum of visually responsive excitatory and inhibitory inputs (Allman et al., 1985). RF size is an important contributor to visual acuity; neurons with large RFs are better at motion decoding and worse at decoding spatial fine structure than neurons with small RFs (Livingstone and Hubel, 1988; Blakemore, 1990; Levitt et al., 2001). RFs are large at birth, and undergo a postnatal refinement process to reach adult size. It has been assumed, based largely on studies of ocular dominance (OD) in primary visual cortex (V1) of cats and monkeys (Hensch et al., 1998; Espinosa and Stryker, 2012), that visual pathway development requires early visual experience during a critical period for maturation but not for maintenance of refined circuitry. Evidence from experiments in both SC (Carrasco et al., 2011; Balmer and Pallas, 2015b) and V1 (e.g., Huang et al., 1999; Fagiolini and Hensch, 2000; van Versendaal et al., 2012) suggests a signaling pathway that involves TrkB receptors. Visual experience activates NMDA receptors, which allow calcium entry into the neuron and activation of CaMKII. This signaling pathway promotes BDNF transcription, leading to TrkB-mediated alterations in GABAergic inhibition (Hong et al., 2008; Lin et al., 2008; Bloodgood et al., 2013; Park and Poo, 2013; Spiegel et al., 2014). This in turn promotes increased inhibition from fast-spiking, GABAergic “basket” type neurons. Mature basket cells and their proteoglycan-rich perineuronal nets (PNNs) enwrap the somata of glutamatergic pyramidal neurons, resulting in reduced plasticity and thus closure of the critical period for ocular dominance plasticity (Bavelier et al., 2010; Beurdeley et al., 2012). Most mammals do not have ocular dominance columns, however, and neither pyramidal nor basket neurons are found outside of the telencephalon (Jones and Hendry, 1984; Peters and Jones, 1984), suggesting that the proposed mechanism may not be generalizable across different brain regions, species, or types of plasticity. An alternative mechanism places maturation of PSD-95-dependent, “silent” synapse maturation as the necessary and sufficient step in critical period plasticity (Huang et al., 2015). PSD-95 anchors glutamate receptors at the postsynaptic density, promoting stability of excitatory glutamatergic synapses in neocortex and hippocampus (Liao et al., 2001; Lüscher and Malenka, 2012).

## Use-dependent plasticity

Excitatory and inhibitory synaptic connections can be made stronger with use and weaker with disuse (Hebb, 1949; Stent, 1973; Quinlan et al., 1999; Philpot et al., 2001; Castillo et al., 2011; Sanes and Kotak, 2011; Trachtenberg, 2015). The threshold for induction of plasticity increases with age (Kirkwood et al., 1995). As a result, use-dependent excitatory and inhibitory plasticity provides flexibility early in life, and stability later. Sensory experience has a powerful influence on the development and plasticity of neural circuits (Munz et al., 2014). Shaping connectivity under the direction of sensory inputs ensures that circuits are tuned to the environment on both developmental and evolutionary time scales. Thus, environmental changes can be incorporated in circuits in an ecologically adaptive way by existing developmental processes.

Not all activity generated in sensory pathways comes from the outside world. Neurons also fire spontaneous action potentials. Spontaneous activity can be highly organized, to the extent that it mimics sensory inputs. Before eye opening, waves of nicotinic acetylcholine-based and then glutamate-based spontaneous activity wash over the retina (Wong et al., 1993; Feller, 2002; Arroyo and Feller, 2016). Due to the retinotopic organization of the visual pathway, these waves will activate neighboring neurons that represent adjacent regions of the visual field. When the eyes open, spontaneous activity declines and is replaced by visually driven activity. Birth and eye opening are uncoupled (Clancy et al., 2001, 2007), however, and exposure to visual experience varies with niche, so some species may rely on visual drive for shaping their visual pathways more than other species.

Whether driven by light or by waves of spontaneous activity, coincident excitation of neighboring neurons that converge on a common target neuron increases the likelihood that the target neuron will fire an action potential. If there are NMDA receptors on the target neuron, there is also a greater likelihood that calcium entry will activate CaMKII and the signaling pathway that leads to insertion of AMPA receptors in the postsynaptic membrane, stabilizing the connections and increasing the synaptic strength (Cline and Constantine-Paton, 1989; Constantine-Paton and Cline, 1998). As a result, even without open eyes, the visual pathway is refined based on the neurons that exhibit the highest degree of cooperative activity (McLaughlin et al., 2003). The question then arises about the relative importance of spontaneous vs. sensory-driven activity in development of sensory pathways.

## Critical periods

**Figure 5:**
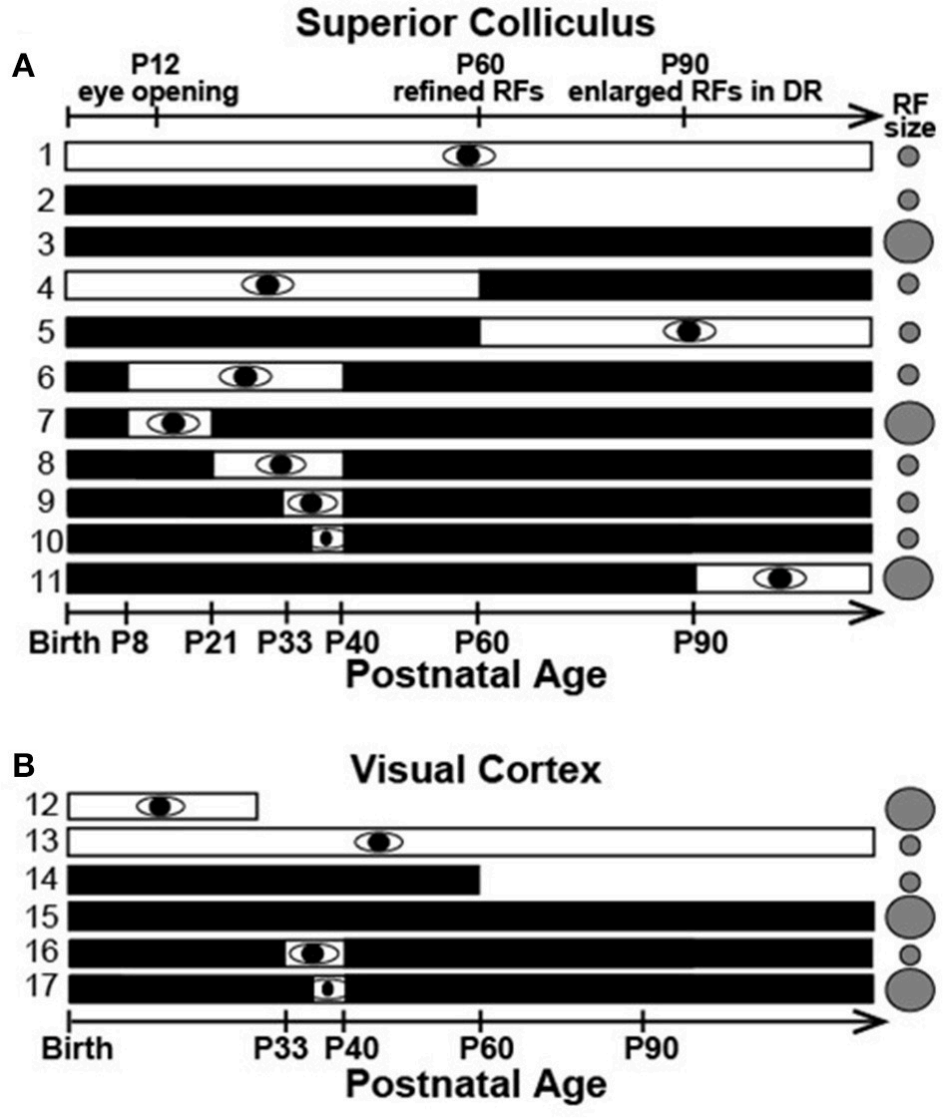
Summary of previous data on timing of RF refinement and sensitivity to visual deprivation. Refinement occurs normally in the dark but late postnatal visual experience is required to maintain adult RFs in both **(A)** SC and **(B)** V1. White and black bars indicate timing of light and dark rearing, respectively. Gray circles indicate adult RF size (Modified from Balmer and Pallas, 2015a, used with permission).

Critical periods allow developing visual circuits to be modified permanently by the environment while providing stable circuitry later in life. Although, spontaneous activity plays an important early role (Kirkby et al., 2013), the dominant view, based largely on studies of ocular dominance plasticity in carnivore and primate visual cortex, contends that visual experience within an early critical period is necessary for maturation and that beyond this period, plasticity is minimal (Espinosa and Stryker, 2012). Our results in hamster SC challenge this view derived from ocular dominance plasticity studies. We find that developmental refinement of visual receptive field (RF) size in both SC and V1 occurs *without visual experience* (Figures 5A_1, 2_,5B_12−14_), but that continued dark rearing results in a loss of RF refinement in adulthood (>P60 days) (Figures 5A_3_,5B_15_; Carrasco et al., 2005; Balmer and Pallas, 2015a). A brief, late juvenile exposure to light stabilizes receptive field size permanently (Figures 5A_6−10_, 5B_16_), but visual experience after postnatal day (P) 60 has no effect (Figure 5A_4, 5, 11_; Carrasco and Pallas, 2006; Balmer and Pallas, 2015a). Interestingly, V1 requires a longer period of late juvenile light exposure to stabilize small RFs than SC (compare Figure 5A_10_ and Figure 5B_16, 17_). These unexpected results refute the hypothesis that subcortical and cortical regions differ in their dependence on vision, and raise the interesting possibility that the current paradigm, derived from classic lab animal models, does not generalize across species, areas, and/or response properties. Other evidence supports this possibility. For example, adult visual cortex is more plastic in mice than cats (Sawtell et al., 2003; Espinosa and Stryker, 2012; Hübener and Bonhoeffer, 2014), and there are species differences in the susceptibility of orientation tuning to early experience. Inhibition is important in gating cortical plasticity in general (Artola and Singer, 1987). It has been proposed that activation of synaptic inhibition in the developing visual cortex is responsible for opening the critical period for ocular dominance plasticity (Hensch et al., 1998; Iwai et al., 2003). Closing it is thought to result from a maturation of GABAergic synapses (Huang et al., 1999; Jiang et al., 2005) that is driven by excitatory inputs (Kuhlman et al., 2013; Gu et al., 2016). Alternatively, there is some evidence for a more critical role of silent synapse maturation in critical period timing (Huang et al., 2015).

## The role of vision in behavior differs among species

The segregation of parallel visual pathways into dorsal “What” and ventral “Where” streams is conserved across primates, carnivores, and rodents (Waleszczyk et al., 2004; Van den Bergh et al., 2010; Wang et al., 2012), but there is tremendous variation across species in the role of vision in survival and behavior (Wilson and Reeder, 2005; Myers et al., 2014; Veilleux and Kirk, 2014). Optics, photoreceptor density, and receptive field size/overlap provide anatomical and physiological limits on acuity (Parker and Hawken, 1985; Troilo et al., 1996; Kaskan et al., 2005; Bleckert et al., 2014). Clearly, species that are more active at night will have limited access to visual information compared to diurnal species. Considerable evidence exists for a linkage between visual acuity and diel activity pattern, with diurnal species having larger eyes/retinae, higher numbers of photoreceptors, and higher visual acuity (Wikler and Rakic, 1996; Veilleux and Kirk, 2014). Species with rapid locomotion, especially predators that rely on sight for prey detection and capture, have larger eyes and higher acuity (Hall et al., 2012). RF size is an important component of pattern vision and object localization, which are arguably more important to survival of prey species than binocular segregation, especially in animals such as rodents that do not have extensive binocular vision (Antonini et al., 1999). Animals with more complex visual behavior, larger visual cortices, and frontally-placed eyes are more likely to have multiple visual representation in cerebral cortex as well as organized submodality representations, such as orientation pinwheels, color blobs, motion tuning modules, ocular dominance columns, etc. (Livingstone and Hubel, 1988; Krubitzer, 2007b; Campi and Krubitzer, 2010; Kaas, 2012; Pallas and Mao, 2012). The collective evidence thus points to strong selective pressure for high-resolution vision in some species, as evidenced in a profound way by these cortical specializations. It is important to examine the role of ecological niche on inter-specific variations in the role of visual experience in receptive field refinement and spatial frequency threshold.

## Variations on a common theme?

Visual behavior and the extent to which animals use visual cues in their behavioral repertoires vary considerably across phyla. Yet most of our knowledge about the functioning of visual pathways comes from species that were selected for their tractability as experimental subjects or for convenience. Early studies of retinal circuitry were initially performed in a wide variety of species, for example salamanders (Werblin and Dowling, 1969), frogs (Barlow, 1953; Lettvin et al., 1959), rabbits (Barlow and Levick, 1965), fish (Witkovsky and Dowling, 1969), and horseshoe crabs (Ratliff and Hartline, 1959) in addition to cats (Kuffler, 1953; Enroth-Cugell and Robson, 1966). David Hubel and Torsten Wiesel used cats and macaque monkeys in their pioneering investigations of developing and adult retinogeniculocortical pathways. These species were chosen with the assumption that what was discovered would be relevant to visual pathway function in infant and adult humans. Since then, there has been an almost wholesale shift toward mice as a model organism for studies of visual system development and plasticity, primarily for the ease of using genetic tools. This has occurred without a full consideration of the behavioral and physiological ecology of mice and possible implications for their visual system organization. Ecological niche is likely to have an important effect on not only the structure and function of the visual pathway in adults, but also on the role of vision in its development. For example, nocturnal, fossorial mammals like mice may depend less on visual experience for visual pathway development than diurnal, cursorial species like primates. This is an important consideration for choosing a model organism for studies of visual system development and plasticity. Furthermore, now that it is becoming easier to manipulate gene expression in a variety of species, mice may lose one reason for their popularity.

## Evidence that the role of sensory experience in development of visual receptive field properties differs between species and between different receptive field properties

The concept of a critical period is firmly embedded in the literature, yet is used in different ways by different investigators. Most use the term to mean an early period of development during which the brain can be modified by the environment, with the implication that after the critical period closes, modification is no longer possible. Some prefer the term “sensitive period” to indicate those developmental events that have a decreased sensitivity to external influence with age, but which can still exhibit some level of experience-dependent modification; that is they are more sensitive to extrinsic influence during a certain time period. Language learning is a good example. It is increasingly becoming apparent, however, that one species critical period is another species sensitive period, making it important to carefully consider which term is used and for what circumstances. As mentioned above, mice can exhibit ocular dominance plasticity as adults, but cats cannot. Does this mean that cats have a critical period but mice have a sensitive period for ocular dominance plasticity? Or that we do not yet know how to demonstrate plasticity in adult cats? The evidence that exercise (Kaneko and Stryker, 2014; Kaneko et al., 2017) and environmental enrichment (Greifzu et al., 2014, 2016) can influence plasticity supports this idea.

The timing of the critical period for ocular dominance plasticity is such that it opens soon after the eyes open and vision becomes possible (Berardi et al., 2000). After it closes, visual acuity does not improve substantially, but whether the potential for ocular dominance plasticity makes increased acuity possible seems unlikely, given that acuity increases in both binocular and non-binocular regions of the visual field. The brain and body size and the evolutionary history of a species is a good predictor of the time course of its brain development (Clancy et al., 2001; Workman et al., 2013), including the time course of its critical/sensitive periods (Berardi et al., 2000). If the same mechanism underlies the opening and closing of these periods across species, then all elements of that mechanism, such as BDNF and its receptor (Huang et al., 1999 and in preparation; Mudd et al., in press), must be in place and operational for different periods of time in different species. In cases, where there are differently timed critical periods for different events within a species, this would also be expected.

Examples of species or regional differences in the relationship between visual experience and development of visual circuitry abound. In mouse retinal ganglion cells, spatiotemporal response properties, and contrast detection thresholds do not require vision for their development, but ON and OFF responses do (Ko et al., 2013; Akimov and Rentería, 2014). Direction selectivity in V1 requires visual experience for even rudimentary development in ferret V1 (Li et al., 2006). It can be modified by experience in cats (Berman and Daw, 1977; Leventhal and Hirsch, 1980) and rats (Fagiolini et al., 1994) but not in mice (Rochefort et al., 2011). Mice, but not rats or cats, exhibit ocular dominance plasticity in adulthood, perhaps because of a different mechanism, or perhaps because in larger animals, adult axons have greater distances to bridge to make new connections (Laing et al., 2015). Dark-rearing has only a modest effect on perceptual (Prusky and Douglas, 2003) and physiological (Kang et al., 2013) acuity in mice, but severely reduces spatial resolution of the X-cell form vision pathway in rat (Fagiolini et al., 1994) and cat visual cortex (Timney et al., 1978; Derrington and Hawken, 1981). Spatial frequency selectivity increases independently of visual experience for up to 3 weeks post-natally in cats, but requires visual experience to improve further (Derrington and Fuchs, 1981; Derrington, 1984). Sensitivity to binocular disparity, a measure of depth perception, increases from birth but does not develop during binocular eyelid suture in cats (Pettigrew, 1974). These various pieces of evidence suggest that species differences in the effects of visual deprivation on development of RF properties do exist, and that even within a species, some RF properties require visual experience and some do not. However, there has been little if any attempt to relate these differences to behavioral ecology or to provide a comprehensive investigation. Thus, comparative studies are essential.

## Visual pathway organization differs between species

Reflecting differences in visual behavior, species also differ markedly in the number and size of visual regions in the brain, and particularly visual cortical areas (Krubitzer, 2007a; Larsen and Krubitzer, 2008; Campi and Krubitzer, 2010). In general, the number and relative size of areas increases across time in mammalian orders, from rodents to carnivores to primates, for example, but within the very large and diverse Order Rodentia, the area devoted to visual cortex correlates with the importance of vision to behavior (Campi and Krubitzer, 2010). Retinal structure and function also varies (Huberman and Niell, 2011). Most rodents have Y/W-ganglion cell-dominated retinae and emphasize the retinocollicular “where” pathway over the X-dominated, retinogeniculocortical “what” pathway that is more dominant in carnivores and primates (Sherman and Spear, 1982; Livingstone and Hubel, 1987; Henderson et al., 1988; Waleszczyk et al., 2004; Li et al., 2015). This difference in specialization of the retinofugal cells is reflected throughout the visual pathway, in the organization of the retina in terms of differences in density and cellular composition from center to periphery, in the presence or absence of eye- and function-specific modules, and in the number of specialized visual cortical areas. Evolution of a nocturnal habit may have required visual adaptations with broad implications (Smale et al., 2003; Ankel-Simons and Rasmussen, 2008). These differences allow categorization into different groups, with rodents likely having different needs for malleability vs. stability of their visual pathways than carnivores or primates. Our current understanding of critical period regulation thus may not fit a variety of species across visual pathway levels. Thus, more attention needs to be paid to the goal of developing an integrated view of visual system development and evolution in mammals.

## Summary

Generation of comparative data is needed to guide *choice of animal models* for visual development studies. Identification of interspecies variations will challenge the *generalizability of mechanistic principles* derived from previous studies of visual development, with the potential to *revise current thinking*. Determining the mechanisms leading to species differences will *provide an answer to the fundamentally important question* of how response properties evolved to match sensory ecology.

## Author contributions

SP wrote the manuscript, based on work done in the author's laboratory as well as work done in other laboratories around the world.

### Conflict of interest statement

The author declares that the research was conducted in the absence of any commercial or financial relationships that could be construed as a potential conflict of interest.
